# (*E*)-*N*′-(9-Anthryl­methyl­idene)-*p*-toluene­sulfono­hydrazide

**DOI:** 10.1107/S160053681003271X

**Published:** 2010-08-21

**Authors:** Abdullah M. Asiri, Mohie E. M. Zayed, Seik Weng Ng

**Affiliations:** a Chemistry Department, Faculty of Science, King Abdul Aziz University, PO Box 80203, Jeddah 21589, Saudi Arabia; b Department of Chemistry, University of Malaya, 50603 Kuala Lumpur, Malaysia

## Abstract

The S—N(H)—N=C linkage in the title mol­ecule, C_22_H_18_N_2_O_2_S, is non-planar [torsion angle = 30.6 (1)°] as the amino N atom is pyramidally coordinated. In the crystal, the amino group acts as a hydrogen-bond donor to an O atom of an adjacent mol­ecule, generating chains running parallel to the *b* axis.

## Related literature

For the structure of the (*E*)-*N*′-benzyl­idene-*p*-toluene­sulf­ono­hydrazide analog, see: Mehrabi *et al.* (2008 ▶).
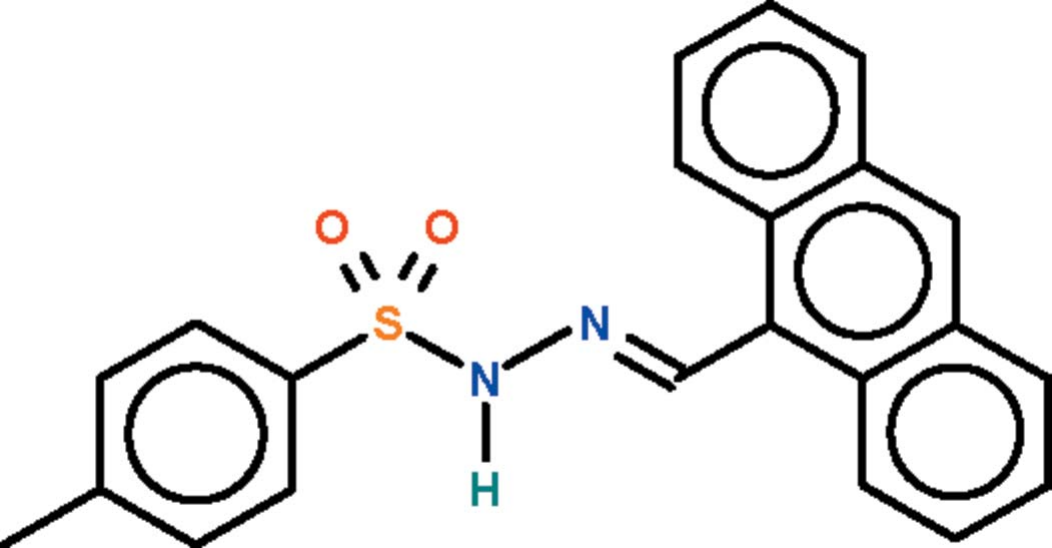


## Experimental

### 

#### Crystal data

C_22_H_18_N_2_O_2_S
*M* *_r_* = 374.44
Orthorhombic, 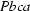

*a* = 17.3634 (15) Å
*b* = 9.2438 (8) Å
*c* = 22.882 (2) Å
*V* = 3672.6 (6) Å^3^
*Z* = 8
Mo *K*α radiation
μ = 0.20 mm^−1^
*T* = 100 K
0.40 × 0.20 × 0.05 mm

#### Data collection

Bruker SMART APEX diffractometer
Absorption correction: multi-scan (*SADABS*; Sheldrick, 1996 ▶) *T*_min_ = 0.926, *T*_max_ = 0.990
22220 measured reflections
4209 independent reflections
3158 reflections with *I* > 2σ(*I*)
*R*_int_ = 0.055

#### Refinement

*R*[*F* ^2^ > 2σ(*F* ^2^)] = 0.040
*wR*(*F* ^2^) = 0.104
*S* = 1.02
4209 reflections
249 parameters
1 restraint
H atoms treated by a mixture of independent and constrained refinement
Δρ_max_ = 0.34 e Å^−3^
Δρ_min_ = −0.46 e Å^−3^

### 

Data collection: *APEX2* (Bruker, 2009 ▶); cell refinement: *SAINT* (Bruker, 2009 ▶); data reduction: *SAINT*; program(s) used to solve structure: *SHELXS97* (Sheldrick, 2008 ▶); program(s) used to refine structure: *SHELXL97* (Sheldrick, 2008 ▶); molecular graphics: *X-SEED* (Barbour, 2001 ▶); software used to prepare material for publication: *publCIF* (Westrip, 2010 ▶).

## Supplementary Material

Crystal structure: contains datablocks global, I. DOI: 10.1107/S160053681003271X/pk2262sup1.cif

Structure factors: contains datablocks I. DOI: 10.1107/S160053681003271X/pk2262Isup2.hkl

Additional supplementary materials:  crystallographic information; 3D view; checkCIF report

## Figures and Tables

**Table 1:** Hydrogen-bond geometry (Å, °)

*D*—H⋯*A*	*D*—H	H⋯*A*	*D*⋯*A*	*D*—H⋯*A*
N1—H1⋯O1^i^	0.86 (1)	2.07 (1)	2.911 (2)	169 (2)

Symmetry code: (i) 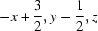.

**Fig. 1.**
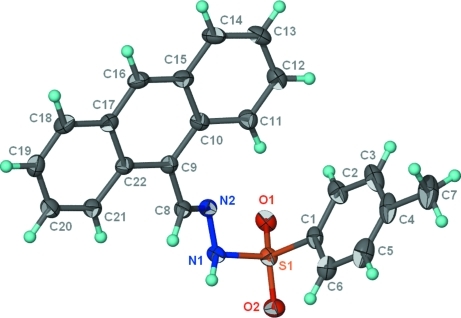
Thermal ellipsoid plot (Barbour, 2001) of C22H18N2O2S at the 70% probability level; hydrogen atoms are drawn as spheres of arbitrary radius.

**Fig. 2.**
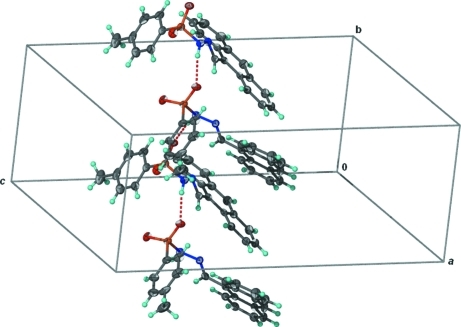
A view of the chain structure resulting from N—H···O hydrogen-bonding.

## supplementary crystallographic information

### Comment

*p*-Toluenesulfonyl hydrazide, CH_3_-4-C_6_H_4_SO_2_NHNH_2_, condenses with carbonyl compounds to form Schiff bases, and among the plethora, nearly a hundred have had their crystal structures determined. The compounds have the azomethine double-bond in an *E*-configuration.  In the Schiff base product between *p*-toluenesulfonyl hydrazide and thiophene-2-carboxaldehyde, the S–N(H)–N═C linkage is non-planar [torsion angle 30.6 (1) °] as the amino nitrogen atom (which bears a hydrogen atom) is pyramidally coordinated (Fig. 1).  The amino group acts as a hydrogen-bond donor to an oxygen atom of an adjacent molecule to generate chains running parallel to the *b*-axis of the cell (Fig. 2).

### Experimental

*p*-Toluenesulfonyl hydrazide (4.66 g, 2.5 mmol) and anthracene-9-carboxaldehyde (5.162.80 g, 2.5 mmol) were heated in methanol (50 ml) for two hours. The cool solution yielded a precipitate that was recrystallized from ethanol and collected in 90% yield.

### Refinement

Carbon-bound H-atoms were placed in calculated positions [C–H 0.95 to 0.99 Å, *U*(H) 1.2 to 1.5*U*_eq_(C)] and were included in the refinement in the riding model approximation. The amino H-atom was located in a difference Fourier map, and was refined with a distance restraint [N–H 0.86 (1) Å]; its temperature factor was freely refined.

### Crystal data

**Table:**

C_22_H_18_N_2_O_2_S	*F*(000) = 1568
*M**_r_* = 374.44	*D*_x_ = 1.354 Mg m^−^^3^
Orthorhombic, *P**b**c**a*	Mo *K*α radiation, λ = 0.71073 Å
Hall symbol: -P 2ac 2ab	Cell parameters from 3593 reflections
*a* = 17.3634 (15) Å	θ = 2.3–27.6°
*b* = 9.2438 (8) Å	µ = 0.20 mm^−^^1^
*c* = 22.882 (2) Å	*T* = 100 K
*V* = 3672.6 (6) Å^3^	Prism, yellow
*Z* = 8	0.40 × 0.20 × 0.05 mm

### Data collection

**Table:**

Bruker SMART APEX diffractometer	4209 independent reflections
Radiation source: fine-focus sealed tube	3158 reflections with *I* > 2σ(*I*)
graphite	*R*_int_ = 0.055
ω scans	θ_max_ = 27.5°, θ_min_ = 1.8°
Absorption correction: multi-scan (*SADABS*; Sheldrick, 1996)	*h* = −18→22
*T*_min_ = 0.926, *T*_max_ = 0.990	*k* = −12→10
22220 measured reflections	*l* = −29→29

### Refinement

**Table:**

Refinement on *F*^2^	Primary atom site location: structure-invariant direct methods
Least-squares matrix: full	Secondary atom site location: difference Fourier map
*R*[*F*^2^ > 2σ(*F*^2^)] = 0.040	Hydrogen site location: inferred from neighbouring sites
*wR*(*F*^2^) = 0.104	H atoms treated by a mixture of independent and constrained refinement
*S* = 1.02	*w* = 1/[σ^2^(*F*_o_^2^) + (0.0442*P*)^2^ + 1.8913*P*] where *P* = (*F*_o_^2^ + 2*F*_c_^2^)/3
4209 reflections	(Δ/σ)_max_ = 0.001
249 parameters	Δρ_max_ = 0.34 e Å^−^^3^
1 restraint	Δρ_min_ = −0.46 e Å^−^^3^

### Fractional atomic coordinates and isotropic or equivalent isotropic displacement parameters (Å^2^)

**Table:**

	*x*	*y*	*z*	*U*_iso_*/*U*_eq_	
S1	0.70021 (2)	0.57688 (5)	0.781940 (19)	0.01816 (12)	
O1	0.67930 (7)	0.71367 (13)	0.75666 (6)	0.0228 (3)	
O2	0.75969 (7)	0.57170 (14)	0.82533 (5)	0.0244 (3)	
N1	0.73235 (8)	0.47229 (17)	0.72874 (6)	0.0188 (3)	
H1	0.7581 (11)	0.4002 (17)	0.7419 (9)	0.032 (6)*	
N2	0.67630 (8)	0.44447 (16)	0.68590 (6)	0.0187 (3)	
C1	0.61574 (10)	0.4956 (2)	0.80808 (8)	0.0207 (4)	
C2	0.54464 (11)	0.5595 (2)	0.79729 (9)	0.0263 (4)	
H2	0.5412	0.6474	0.7760	0.032*	
C3	0.47881 (11)	0.4919 (2)	0.81839 (9)	0.0324 (5)	
H3	0.4299	0.5341	0.8111	0.039*	
C4	0.48310 (12)	0.3638 (2)	0.84988 (9)	0.0319 (5)	
C5	0.55497 (13)	0.3014 (2)	0.85908 (9)	0.0326 (5)	
H5	0.5584	0.2129	0.8800	0.039*	
C6	0.62165 (11)	0.3659 (2)	0.83825 (8)	0.0256 (4)	
H6	0.6705	0.3220	0.8445	0.031*	
C7	0.41088 (13)	0.2933 (3)	0.87297 (10)	0.0437 (6)	
H7A	0.3755	0.3679	0.8872	0.066*	
H7B	0.4242	0.2279	0.9051	0.066*	
H7C	0.3861	0.2382	0.8416	0.066*	
C8	0.68260 (10)	0.32154 (19)	0.66045 (7)	0.0190 (4)	
H8	0.7224	0.2580	0.6727	0.023*	
C9	0.63107 (10)	0.27431 (19)	0.61321 (7)	0.0181 (4)	
C10	0.55246 (10)	0.31693 (19)	0.61075 (8)	0.0196 (4)	
C11	0.51458 (10)	0.3970 (2)	0.65569 (8)	0.0225 (4)	
H11	0.5434	0.4292	0.6885	0.027*	
C12	0.43788 (11)	0.4284 (2)	0.65260 (9)	0.0256 (4)	
H12	0.4139	0.4789	0.6839	0.031*	
C13	0.39353 (11)	0.3870 (2)	0.60350 (9)	0.0284 (4)	
H13	0.3406	0.4124	0.6013	0.034*	
C14	0.42673 (11)	0.3113 (2)	0.55987 (9)	0.0274 (4)	
H14	0.3968	0.2849	0.5268	0.033*	
C15	0.50621 (10)	0.2698 (2)	0.56239 (8)	0.0232 (4)	
C16	0.53801 (11)	0.1800 (2)	0.51995 (8)	0.0248 (4)	
H16	0.5071	0.1511	0.4877	0.030*	
C17	0.61374 (10)	0.1311 (2)	0.52330 (8)	0.0207 (4)	
C18	0.64417 (11)	0.0329 (2)	0.48093 (8)	0.0249 (4)	
H18	0.6121	0.0005	0.4500	0.030*	
C19	0.71804 (11)	−0.0149 (2)	0.48399 (8)	0.0247 (4)	
H19	0.7372	−0.0808	0.4557	0.030*	
C20	0.76612 (11)	0.0344 (2)	0.52973 (8)	0.0229 (4)	
H20	0.8179	0.0017	0.5316	0.028*	
C21	0.73965 (10)	0.1276 (2)	0.57109 (8)	0.0204 (4)	
H21	0.7736	0.1594	0.6010	0.025*	
C22	0.66168 (10)	0.17906 (19)	0.57053 (7)	0.0184 (4)	

### Atomic displacement parameters (Å^2^)

**Table:**

	*U*^11^	*U*^22^	*U*^33^	*U*^12^	*U*^13^	*U*^23^
S1	0.0166 (2)	0.0169 (2)	0.0210 (2)	−0.00194 (17)	0.00059 (17)	−0.00158 (17)
O1	0.0222 (7)	0.0163 (6)	0.0300 (7)	−0.0008 (5)	0.0016 (5)	0.0003 (5)
O2	0.0203 (7)	0.0287 (7)	0.0241 (7)	−0.0025 (6)	−0.0044 (5)	−0.0040 (6)
N1	0.0164 (7)	0.0189 (8)	0.0211 (8)	0.0014 (6)	−0.0012 (6)	−0.0008 (6)
N2	0.0167 (7)	0.0209 (8)	0.0184 (7)	−0.0022 (6)	−0.0009 (6)	−0.0003 (6)
C1	0.0204 (9)	0.0205 (9)	0.0212 (9)	−0.0052 (7)	0.0050 (7)	−0.0043 (7)
C2	0.0219 (9)	0.0249 (10)	0.0323 (10)	−0.0008 (8)	0.0037 (8)	−0.0067 (8)
C3	0.0222 (10)	0.0366 (12)	0.0386 (12)	−0.0036 (9)	0.0077 (9)	−0.0149 (10)
C4	0.0330 (11)	0.0353 (12)	0.0274 (10)	−0.0164 (9)	0.0122 (9)	−0.0173 (9)
C5	0.0449 (13)	0.0250 (11)	0.0279 (10)	−0.0115 (9)	0.0087 (9)	−0.0030 (9)
C6	0.0296 (10)	0.0220 (10)	0.0252 (9)	−0.0029 (8)	0.0049 (8)	−0.0017 (8)
C7	0.0381 (13)	0.0554 (16)	0.0375 (12)	−0.0261 (11)	0.0144 (10)	−0.0162 (11)
C8	0.0177 (9)	0.0198 (9)	0.0195 (9)	0.0003 (7)	0.0016 (7)	0.0035 (7)
C9	0.0190 (9)	0.0171 (9)	0.0182 (8)	−0.0017 (7)	0.0004 (7)	0.0026 (7)
C10	0.0187 (9)	0.0184 (9)	0.0216 (9)	−0.0010 (7)	−0.0008 (7)	0.0031 (7)
C11	0.0197 (9)	0.0241 (10)	0.0238 (9)	−0.0020 (7)	0.0005 (7)	−0.0015 (8)
C12	0.0220 (9)	0.0249 (10)	0.0300 (10)	0.0017 (8)	0.0035 (8)	−0.0020 (8)
C13	0.0184 (9)	0.0299 (11)	0.0369 (11)	0.0042 (8)	−0.0030 (8)	0.0015 (9)
C14	0.0229 (10)	0.0311 (11)	0.0282 (10)	0.0044 (8)	−0.0083 (8)	−0.0004 (9)
C15	0.0213 (9)	0.0248 (10)	0.0235 (9)	0.0015 (8)	−0.0036 (7)	0.0035 (8)
C16	0.0243 (10)	0.0297 (10)	0.0203 (9)	0.0018 (8)	−0.0067 (8)	−0.0007 (8)
C17	0.0222 (9)	0.0218 (9)	0.0182 (8)	0.0009 (7)	−0.0005 (7)	0.0022 (7)
C18	0.0274 (10)	0.0283 (10)	0.0191 (9)	−0.0004 (8)	−0.0040 (8)	−0.0010 (8)
C19	0.0289 (10)	0.0252 (10)	0.0200 (9)	0.0015 (8)	0.0039 (8)	−0.0007 (8)
C20	0.0199 (9)	0.0231 (10)	0.0258 (10)	0.0020 (7)	0.0023 (7)	0.0031 (8)
C21	0.0193 (9)	0.0210 (9)	0.0210 (8)	−0.0010 (7)	0.0005 (7)	0.0019 (7)
C22	0.0199 (9)	0.0176 (9)	0.0176 (8)	−0.0018 (7)	0.0001 (7)	0.0046 (7)

### Geometric parameters (Å, °)

**Table:**

S1—O1	1.4370 (13)	C9—C10	1.422 (2)
S1—O2	1.4334 (13)	C10—C11	1.428 (2)
S1—N1	1.6516 (15)	C10—C15	1.435 (2)
S1—C1	1.7532 (18)	C11—C12	1.365 (3)
N1—N2	1.405 (2)	C11—H11	0.9500
N1—H1	0.857 (9)	C12—C13	1.415 (3)
N2—C8	1.281 (2)	C12—H12	0.9500
C1—C6	1.387 (3)	C13—C14	1.349 (3)
C1—C2	1.391 (3)	C13—H13	0.9500
C2—C3	1.389 (3)	C14—C15	1.434 (3)
C2—H2	0.9500	C14—H14	0.9500
C3—C4	1.388 (3)	C15—C16	1.392 (3)
C3—H3	0.9500	C16—C17	1.392 (3)
C4—C5	1.391 (3)	C16—H16	0.9500
C4—C7	1.509 (3)	C17—C18	1.429 (3)
C5—C6	1.387 (3)	C17—C22	1.434 (2)
C5—H5	0.9500	C18—C19	1.358 (3)
C6—H6	0.9500	C18—H18	0.9500
C7—H7A	0.9800	C19—C20	1.414 (3)
C7—H7B	0.9800	C19—H19	0.9500
C7—H7C	0.9800	C20—C21	1.360 (2)
C8—C9	1.470 (2)	C20—H20	0.9500
C8—H8	0.9500	C21—C22	1.435 (2)
C9—C22	1.418 (2)	C21—H21	0.9500
			
O1—S1—O2	119.35 (8)	C9—C10—C11	123.88 (16)
O1—S1—N1	107.68 (8)	C9—C10—C15	118.93 (16)
O2—S1—N1	104.33 (8)	C11—C10—C15	117.07 (16)
O1—S1—C1	107.65 (8)	C12—C11—C10	121.50 (18)
O2—S1—C1	110.62 (8)	C12—C11—H11	119.3
N1—S1—C1	106.45 (8)	C10—C11—H11	119.3
N2—N1—S1	112.79 (11)	C11—C12—C13	120.98 (18)
N2—N1—H1	117.7 (15)	C11—C12—H12	119.5
S1—N1—H1	111.8 (14)	C13—C12—H12	119.5
C8—N2—N1	114.84 (15)	C14—C13—C12	119.71 (17)
C6—C1—C2	121.41 (17)	C14—C13—H13	120.1
C6—C1—S1	118.58 (14)	C12—C13—H13	120.1
C2—C1—S1	120.00 (15)	C13—C14—C15	121.36 (18)
C3—C2—C1	118.52 (19)	C13—C14—H14	119.3
C3—C2—H2	120.7	C15—C14—H14	119.3
C1—C2—H2	120.7	C16—C15—C10	119.80 (16)
C4—C3—C2	121.3 (2)	C16—C15—C14	120.89 (17)
C4—C3—H3	119.3	C10—C15—C14	119.26 (17)
C2—C3—H3	119.3	C17—C16—C15	122.00 (17)
C3—C4—C5	118.72 (18)	C17—C16—H16	119.0
C3—C4—C7	120.4 (2)	C15—C16—H16	119.0
C5—C4—C7	120.9 (2)	C16—C17—C18	121.19 (17)
C6—C5—C4	121.3 (2)	C16—C17—C22	119.28 (17)
C6—C5—H5	119.4	C18—C17—C22	119.52 (16)
C4—C5—H5	119.4	C19—C18—C17	121.39 (17)
C5—C6—C1	118.73 (19)	C19—C18—H18	119.3
C5—C6—H6	120.6	C17—C18—H18	119.3
C1—C6—H6	120.6	C18—C19—C20	119.38 (17)
C4—C7—H7A	109.5	C18—C19—H19	120.3
C4—C7—H7B	109.5	C20—C19—H19	120.3
H7A—C7—H7B	109.5	C21—C20—C19	121.31 (17)
C4—C7—H7C	109.5	C21—C20—H20	119.3
H7A—C7—H7C	109.5	C19—C20—H20	119.3
H7B—C7—H7C	109.5	C20—C21—C22	121.52 (17)
N2—C8—C9	123.06 (16)	C20—C21—H21	119.2
N2—C8—H8	118.5	C22—C21—H21	119.2
C9—C8—H8	118.5	C9—C22—C21	123.60 (16)
C22—C9—C10	120.31 (16)	C9—C22—C17	119.55 (16)
C22—C9—C8	117.56 (15)	C21—C22—C17	116.85 (16)
C10—C9—C8	122.09 (16)		
			
O1—S1—N1—N2	−62.00 (13)	C10—C11—C12—C13	−2.3 (3)
O2—S1—N1—N2	170.23 (11)	C11—C12—C13—C14	2.1 (3)
C1—S1—N1—N2	53.21 (14)	C12—C13—C14—C15	0.9 (3)
S1—N1—N2—C8	−149.39 (13)	C9—C10—C15—C16	2.1 (3)
O1—S1—C1—C6	−175.60 (14)	C11—C10—C15—C16	−174.03 (17)
O2—S1—C1—C6	−43.59 (17)	C9—C10—C15—C14	179.46 (17)
N1—S1—C1—C6	69.17 (16)	C11—C10—C15—C14	3.4 (3)
O1—S1—C1—C2	5.81 (18)	C13—C14—C15—C16	173.73 (19)
O2—S1—C1—C2	137.82 (15)	C13—C14—C15—C10	−3.6 (3)
N1—S1—C1—C2	−109.42 (16)	C10—C15—C16—C17	1.3 (3)
C6—C1—C2—C3	1.2 (3)	C14—C15—C16—C17	−176.08 (18)
S1—C1—C2—C3	179.71 (15)	C15—C16—C17—C18	176.73 (18)
C1—C2—C3—C4	0.3 (3)	C15—C16—C17—C22	−2.6 (3)
C2—C3—C4—C5	−1.4 (3)	C16—C17—C18—C19	179.93 (18)
C2—C3—C4—C7	179.28 (18)	C22—C17—C18—C19	−0.8 (3)
C3—C4—C5—C6	1.0 (3)	C17—C18—C19—C20	−0.6 (3)
C7—C4—C5—C6	−179.68 (18)	C18—C19—C20—C21	0.7 (3)
C4—C5—C6—C1	0.4 (3)	C19—C20—C21—C22	0.6 (3)
C2—C1—C6—C5	−1.6 (3)	C10—C9—C22—C21	−177.99 (16)
S1—C1—C6—C5	179.88 (14)	C8—C9—C22—C21	−0.2 (3)
N1—N2—C8—C9	−178.04 (15)	C10—C9—C22—C17	2.8 (3)
N2—C8—C9—C22	149.92 (17)	C8—C9—C22—C17	−179.43 (15)
N2—C8—C9—C10	−32.4 (3)	C20—C21—C22—C9	178.91 (17)
C22—C9—C10—C11	171.73 (17)	C20—C21—C22—C17	−1.9 (3)
C8—C9—C10—C11	−5.9 (3)	C16—C17—C22—C9	0.5 (3)
C22—C9—C10—C15	−4.1 (3)	C18—C17—C22—C9	−178.81 (17)
C8—C9—C10—C15	178.27 (16)	C16—C17—C22—C21	−178.75 (17)
C9—C10—C11—C12	−176.38 (18)	C18—C17—C22—C21	1.9 (2)
C15—C10—C11—C12	−0.5 (3)		

### Hydrogen-bond geometry (Å, °)

**Table:**

*D*—H···*A*	*D*—H	H···*A*	*D*···*A*	*D*—H···*A*
N1—H1···O1^i^	0.86 (1)	2.07 (1)	2.911 (2)	169 (2)

Symmetry codes: (i) −*x*+3/2, *y*−1/2, *z*.
